# Stress-Induced Microstructural Alterations Correlate With the Cognitive Performance of Rats: A Longitudinal *in vivo* Diffusion Tensor Imaging Study

**DOI:** 10.3389/fnins.2020.00474

**Published:** 2020-06-03

**Authors:** Szilvia Anett Nagy, Anett Vranesics, Zsófia Varga, Dávid Csabai, Nóra Bruszt, Zsolt Kristóf Bali, Gábor Perlaki, István Hernádi, Zoltán Berente, Attila Miseta, Tamás Dóczi, Boldizsár Czéh

**Affiliations:** ^1^Neurobiology of Stress Research Group, Szentágothai Research Centre, University of Pécs, Pécs, Hungary; ^2^MTA-PTE, Clinical Neuroscience MR Research Group, Pécs, Hungary; ^3^Department of Neurosurgery, Medical School, University of Pécs, Pécs, Hungary; ^4^Pécs Diagnostic Centre, Pécs, Hungary; ^5^Department of Laboratory Medicine, Medical School, University of Pécs, Pécs, Hungary; ^6^Research Group for Experimental Diagnostic Imaging, Medical School, University of Pécs, Pécs, Hungary; ^7^Department of Biochemistry and Medical Chemistry, Medical School, University of Pécs, Pécs, Hungary; ^8^Translational Neuroscience Research Group, Centre for Neuroscience, Szentágothai Research Centre, University of Pécs, Pécs, Hungary; ^9^Department of Physiology, Medical School, University of Pécs, Pécs, Hungary; ^10^Grastyán Translational Research Centre, University of Pécs, Pécs, Hungary; ^11^Department of Experimental Zoology and Neurobiology, Faculty of Sciences, University of Pécs, Pécs, Hungary

**Keywords:** chronic stress, magnetic resonance imaging, DTI, fractional anisotropy, mean diffusivity, radial diffusivity, Morris water maze, novel object recognition test

## Abstract

**Background:** Stress-induced cellular changes in limbic brain structures contribute to the development of various psychopathologies. *In vivo* detection of these microstructural changes may help us to develop objective biomarkers for psychiatric disorders. Diffusion tensor imaging (DTI) is an advanced neuroimaging technique that enables the non-invasive examination of white matter integrity and provides insights into the microstructure of pathways connecting brain areas.

**Objective:** Our aim was to examine the temporal dynamics of stress-induced structural changes with repeated *in vivo* DTI scans and correlate them with behavioral alterations.

**Methods:** Out of 32 young adult male rats, 16 were exposed to daily immobilization stress for 3 weeks. Four DTI measurements were done: one before the stress exposure (*baseline*), two scans during the stress (*acute* and *chronic* phases), and a last one 2 weeks after the end of the stress protocol (*recovery*). We used a 4.7T small-animal MRI system and examined 18 gray and white matter structures calculating the following parameters: fractional anisotropy (FA), mean diffusivity (MD), axial diffusivity (AD), and radial diffusivity (RD). T2-weighted images were used for volumetry. Cognitive performance and anxiety levels of the animals were assessed in the Morris water maze, novel object recognition, open field, and elevated plus maze tests.

**Results:** Reduced FA and increased MD and RD values were found in the corpus callosum and external capsule of stressed rats. Stress increased RD in the anterior commissure and reduced MD and RD in the amygdala. We observed time-dependent changes in several DTI parameters as the rats matured, but we found no evidence of stress-induced volumetric alterations in the brains. Stressed rats displayed cognitive impairments and we found numerous correlations between the cognitive performance of the animals and between various DTI metrics of the inferior colliculus, corpus callosum, anterior commissure, and amygdala.

**Conclusions:** Our data provide further support to the translational value of DTI studies and suggest that chronic stress exposure results in similar white matter microstructural alterations that have been documented in stress-related psychiatric disorders. These DTI findings imply microstructural abnormalities in the brain, which may underlie the cognitive deficits that are often present in stress-related mental disorders.

## Introduction

Stress is an important element of our everyday life as all living organisms need to overcome external and internal challenges to succeed in life. However, when stress is too severe, or when it becomes chronic, then it may lead to the development of various somatic and mental disorders (McEwen, 1998; Kendler et al., 1999; Chandola et al., 2006; Dube et al., 2009; Lanius et al., 2010; Steptoe and Kivimaki, 2012). It is well-documented that chronic stress can induce morphological and functional changes of specific limbic brain areas, and these alterations are believed to contribute to the development of various psychopathologies (Pittenger and Duman, 2008; MacQueen and Frodl, 2011; Popoli et al., 2011). Numerous postmortem histopathological studies have documented that stress can modify the dendritic architecture of pyramidal neurons, inhibits adult hippocampal neurogenesis, and affects glial cells, as well as GABAergic interneurons (Lucassen et al., 2014; McEwen et al., 2015, 2016; Fogaca and Duman, 2019). These neuroanatomical alterations contribute to the disturbed functioning of synaptic contacts (Popoli et al., 2011), which in turn leads to disrupted structural and functional connectivity of neuronal networks and eventually results in impaired emotional and cognitive functioning (de Kloet et al., 1999; Evans and Schamberg, 2009; Kim et al., 2013; Sousa, 2016; Duman et al., 2019).

The rapid methodological developments in magnetic resonance imaging (MRI) allows us to investigate the stress-induced structural changes directly in living organisms. This opportunity inspired numerous research groups to examine the impact of chronic stress on brain activity and morphology in living animals. Henckens and co-workers were the first to carry out a detailed investigation of the consequences of 10 days of repeated immobilization stress on the structural integrity and functional connectivity patterns in the rodent brain, using high-resolution structural MRI, diffusion kurtosis imaging, and resting-state functional MRI (Henckens et al., 2015). They reported that chronic stress exposure can alter large-scale functional connectivity networks by increasing connectivity in the somatosensory, visual, and default mode networks, but it does not induce any major changes in gray matter volumes of the rat brain (Henckens et al., 2015). Later studies, however, found hippocampal atrophy in rats subjected to a 4-weeks chronic unpredictable mild stress paradigm (Li et al., 2017). More recently, a longitudinal neuroimaging study examined the effects of chronic unpredictable stress on the structure and functional connectome of the rat brain in stress-susceptible and stress-resilient animals (Magalhaes et al., 2018). They found stress-induced structural atrophy of several limbic and non-limbic brain areas, which was associated with increased functional connectivity in a network formed by these specific regions (Magalhaes et al., 2018).

Overall, these MRI studies confirm and further extend the earlier histopathological findings documenting disrupted connectivity between key limbic structures that are known to regulate the stress response. Diffusion tensor imaging (DTI) is an MRI-based neuroimaging technique, which enables the examination of white (and gray) matter integrity and provides insights into the microstructure of pathways connecting different brain areas. The typical readouts of DTI studies are mean diffusivity (MD) and fractional anisotropy (FA), which represent the overall diffusion regardless of directionality (i.e., the degree to which tissue organization limits the diffusion of water molecules) and the degree of diffusion anisotropy (i.e., directionality of diffusion related to tract integrity and the alignment of neuronal fibers), respectively. Other DTI-related parameters, such as axial diffusivity (AD) and radial diffusivity (RD), indicate axonal, and myelin microstructural changes. Therefore, developmental alterations (e.g., myelination), fiber organization, as well as structural integrity of the white matter can be detected by DTI (Yoshida et al., 2013). More recently, advanced diffusion-weighted imaging techniques were developed, such as high angular resolution diffusion (including diffusion spectrum and q-ball imaging) and diffusion kurtosis imaging to model diffusion signal more precisely (Tuch, 2004; Wedeen et al., 2005; Jensen and Helpern, 2010). However, these advanced methods typically require substantial increase in image acquisition time compared to DTI and therefore cannot be properly applied for *in vivo* animal studies. Consequently, DTI is by far the most commonly used method to characterize the microstructural changes affecting white and gray matter areas.

So far, only a handful of studies have used diffusion MRI to examine structural alterations in the brains of experimental animals exposed to chronic stress, and the outcome of these studies are inconsistent. Delgado y Palacios and co-workers were the first to report on subtle substructural changes in the hippocampus of chronically stressed rats using *in vivo* diffusion kurtosis imaging (Delgado y Palacios et al., 2011). Later, the same research group investigated diffusion properties in the prefrontal cortex, caudate putamen, and amygdala and found that mean kurtosis in the striatum was significantly different between the stress-susceptible and stress-resilient animals (Delgado y Palacios et al., 2014). Parallel to these findings, Vestergaard-Poulsen et al. conducted a high-field (16.4 T) diffusion-weighted MRI in combination with quantitative biophysical modeling of the hippocampal dendritic loss using rats subjected to 3 weeks of restraint stress and found that diffusion-weighted MRI data could sensitively detect regional dendritic loss (Vestergaard-Poulsen et al., 2011). Another research group found significant changes in MD, FA, AD, and RD values in numerous brain areas suggesting demyelination and axonal damage (Hemanth Kumar et al., 2014). Yet, another research group found no evidence for white matter microstructural changes in rats exposed to 10 days of repeated immobilization stress (Henckens et al., 2015). Another study using a tract-based spatial statistics analysis approach reported that stress can increase FA and reduced MD and RD in several white matter bundles of the brain after 2 weeks of repeated inescapable stress (Magalhaes et al., 2017). Others reported increased FA in the hypothalamus and hippocampal CA3 in stress-susceptible mice after 10 days of social defeat stress (Anacker et al., 2016). *Ex-vivo* diffusion MRI and diffusion kurtosis imaging studies documented specific microstructural changes in the hippocampus, amygdala, and several cortical areas of rats exposed to chronic stress (Khan et al., 2016a,b, 2018). These findings have been confirmed by a recent *in vivo* imaging experiment (Liu X. et al., 2018).

**Figure 1:**
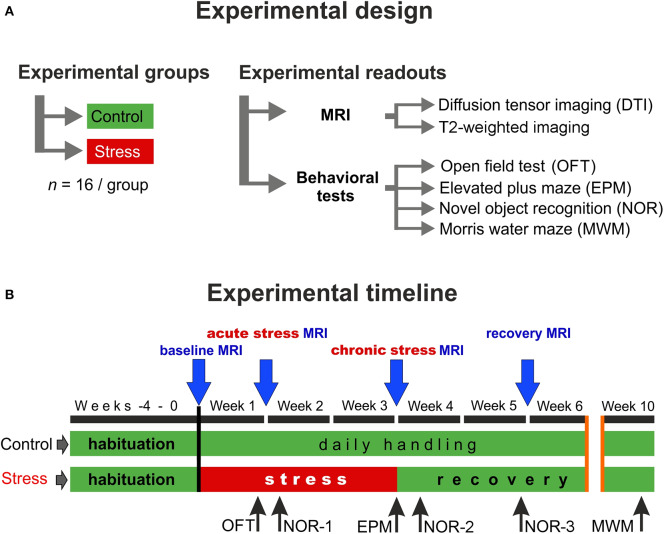
Experimental design and the timeline of the procedures. **(A)** In this study, two groups of young adult male Sprague–Dawley rats were used (*n* = 16 controls and *n* = 16 stressed animals). We did repeated *in vivo* MR imaging and detailed behavioral profiling to assess the effects of chronic stress. **(B)** MRI measurements were performed on four occasions: first before the stress (*baseline*), second during the 1st week of the stress exposure (*acute* stress effect), the third one on the last week of the stress (*chronic* stress effect), and the last one after 2 weeks of recovery period (*recovery* effect). We used various behavioral tests to assess the emotional and cognitive status of the animals. Blue arrows indicate the timing of MRI scans and the black arrows specify the behavioral tests.

To address these discrepancies, we designed an experiment to examine the temporal dynamics of the stress response using repeated DTI measurements of rats subjected to 3 weeks of daily restraint stress. Our hypothesis was that we should observe different microstructural parameters before, during, and after the stress exposure and that the DTI results should be able to differentiate between the *acute* and *chronic* phases of the stress response. We used a chronic stress paradigm, which is known to be stressful for rodents and reliably induce structural changes in the hippocampus and prefrontal cortex including dendritic atrophy of pyramidal cells and reduced neurogenesis in the adult dentate gyrus (Cook and Wellman, 2004; Radley et al., 2004; McLaughlin et al., 2007; Perez-Cruz et al., 2009a,b; Veena et al., 2009). We carried out repeated DTI measurements at four time points: once before the stress, twice during the stress procedures, and a last one after a recovery period of 2 weeks (Figure 1). In addition, through behavioral profiling, we extensively assessed the effects of chronic stress on the animals' cognitive capacity and anxiety levels as numerous studies have documented that these behavioral outcomes are influenced by chronic stress exposure (Katz et al., 1981; Baker and Kim, 2002; Bowman et al., 2003; Gouirand and Matuszewich, 2005; Rygula et al., 2005; Bondi et al., 2008). The cognitive performance of the animals was assessed in the Morris water maze and novel object recognition tests and we conducted elevated plus maze and open field tests to assess anxiety levels. Our hypothesis was that stress should influence the behavior and that the DTI results should correlate with the behavioral performance of the animals.

## Materials and Methods

### Ethics

All animal procedures were carried out in accordance with the guidelines of Decree No. 40/2013 (II. 14) of the Hungarian Government and the EU Directive 2010/63/EU. The experiments were approved by the Hungarian Ethical Committee on Animal Research according to the Ethical Codex of Animal Experiments (License No. BA02/2000-12/2015). Throughout the entire experiment, adequate measures were taken to minimize pain, or discomfort for the experimental animals.

### Animals

Thirty-two young male Sprague–Dawley rats (Charles River Laboratories, Sulzfeld, Germany) aged 4 weeks (50–80 *g*) upon arrival were group housed in plastic cages (378 × 217 × 180 mm, equipped with feeder and bottle container) under standard animal room conditions (temperature 22–24°C; humidity 45–55%; 12 h light/dark cycle; milled chow and water *ad-libitum*).

### Experimental Design

The experimental design and the timeline of the procedures are depicted in Figure 1. First, the animals were allowed to habituate to the new housing conditions for 4 weeks. During this habituation period and throughout the entire experiment, all animals were handled daily. After the 4-weeks habituation period, when animals were 8 weeks old young adults, they were randomly selected and divided into two groups: the control group (*n* = 16) contained animals that were left completely undisturbed, while the stress group (*n* = 16) received daily restraint stress for 21 days (Figure 1A). MRI measurements were performed at four time points: the first scan was done before the stress procedure (*baseline* measurements), the second scan was carried out 7 days after the beginning of the stress exposure (*acute* stress effect), the 3rd scan was done at the end of the 3-weeks stress protocol (*chronic* stress effect), and the last scan was done 2 weeks after the end of stress procedures (*recovery* period) (Figure 1B).

### Restraint Stress Procedures

Restraint stress protocol was used since it has been demonstrated that this is stressful for rodents and results in pronounced structural changes in the hippocampus and prefrontal cortex including dendritic atrophy and reduced neurogenesis in the adult dentate gyrus (Cook and Wellman, 2004; Radley et al., 2004; McLaughlin et al., 2007; Perez-Cruz et al., 2009a,b; Veena et al., 2009). During the restraint stress protocol, the animals were immobilized daily for 6 h, between 07:00 a.m. and 1:00 p.m. using well-ventilated acrylic tubes (Harvard Apparatus, USA) in accordance with our previous protocol (Perez-Cruz et al., 2009a,b). Control rats were kept in a different room and were not subjected to any kind of stress except daily handling.

### *In vivo* MRI

All acquisitions were performed using a 4.7T small-animal MRI system running Paravision 6.0.1 (Pharmascan 47/16 US; Bruker BioSpin MRI GmbH, Ettlingen, Germany) with a gradient strength of 380 mT/m and a slew rate of 3,420 T/m/s, a circularly polarized hydrogen transmit only volume coil (outer/inner diameter = 89/72 mm), and a circularly polarized hydrogen receive only surface coil anatomically shaped for rat brain. MRI measurements were performed under inhalation anesthesia using 1.8–2.5% (3% for induction) isoflurane (Forane; Abbvie, Budapest) in 1:2 mixture of O_2_/N_2_O, administered via a nosecone. Each rat was placed in prone position on a heated water pad to maintain rectal temperature at ~37°C, while a head holder with ear and bite bars were used to prevent head motion. Respiration was monitored using a pressure sensor placed below the abdomen (SA Instruments, Inc., Stony Brook, NY, USA) and was stable at the range of 50–60 breaths/min under anesthesia.

After a gradient-echo localizer scan in three directions, the imaging protocol included fat-suppressed T2-weighted two dimensional fast-spin echo imaging (2D RARE) in axial, sagittal and coronal planes. Axial T2-weighted images were used for volumetric purposes with the following parameters: TR = 2,429 ms; TE = 36 ms; echo spacing = 12 ms; echo train length = 8; field of view (FOV) = 35 × 35 mm^2^; matrix = 256 × 256; slice thickness = 0.7 mm; interslice gap = 0.3 mm.

In order to optimize B0 field homogeneity, DTI was performed after field map-based shimming using Bruker MAPSHIM protocol. Fat-suppressed DTI data were obtained using a four-shot segmented spin-echo echo planar imaging sequence with 30 diffusion gradient directions sampled on a half sphere (TR = 2,000 ms; TE = 31.35 ms; *b*-value = 1,000 s/mm^2^, five reference images with no diffusion gradients applied; diffusion gradient's duration and separation = 4.3 and 10.5 ms, respectively, number of averages = 2; 15 axial slices; FOV = 30 × 30 mm^2^; matrix = 240 × 240; slice thickness = 1 mm; interslice gap = 0.2 mm).

### Data Analysis of the MR Imaging

#### Preprocessing

After careful inspection of the acquired MR data, a few animals had to be excluded due to considerable motion artifacts. In total, 27 rats (*n* = 13 stressed and *n* = 14 controls) were used for diffusion analysis and 26 animals (*n* = 12 stressed and *n* = 14 controls) were involved in MR volumetry.

In order to analyze volumetric and diffusion-related alterations, MRI data were first converted from Bruker format to NIfTI using a Python script. The voxel size of the images was scaled up individually by the factor of 10 to better approximate human dimensions. Then, to improve image registration and segmentation, Brain Extraction Tool provided by FMRIB Software Library (FSL) was applied on the raw MRI data to eliminate non-brain tissues including skull, skin, fat, muscles, and other surrounding tissues (Smith, 2002). Here, the fractional intensity threshold was set to 0.65 and the coordinates (in voxels) for center of initial brain surface sphere were individually chosen to further improve brain extraction. Skull stripping errors were manually corrected when it was necessary by FSLview.

The CUDA implementation of FSL eddy (eddy_cuda7.0) was used to correct diffusion data for susceptibility-induced distortions, eddy currents, and subject motion, and to perform positive and negative outlier detection and replacement for slices with average intensity at least two standard deviations lower than expected (Andersson and Sotiropoulos, 2016; Andersson et al., 2016). Using FMRIB's diffusion toolbox (FDT v3.0, https://fsl.fmrib.ox.ac.uk/fsl/fslwiki/FDT), DTIFIT was applied to fit a single tensor model at each voxel of the preprocessed diffusion-weighted data and to calculate maps of FA, MD, eigenvalues (L_1,2,3_), and eigenvectors (V_1,2,3_).

#### Diffusion Analysis

To evaluate diffusion alterations, 18 gray and white matter structures were segmented based on a brain-extracted three-dimensional DTI rat brain atlas (Rumple et al., 2013) using the following steps:

1. Brain-extracted FA image was registered to atlas space (seven degrees-of-freedom linear fit) using FMRIB's Linear Image Registration Tool (Jenkinson and Smith, 2001) and sinc interpolation.
2. The inverse of the spatial transformation from diffusion space to atlas space was applied to align the 18 brain masks to native diffusion space, where diffusion analyses were performed.

**Figure 2:**
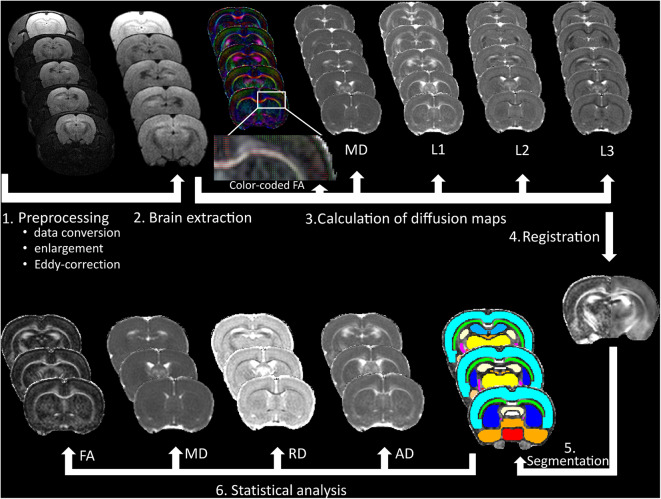
Data processing of the DTI measurements. After pre-processing of the raw data, (1) brain extraction was made to calculate fractional anisotropy (FA), mean diffusivity (MD), first (L1), second (L2), and third (L3) eigenvalues and (2) to improve registration accuracy to a rat brain atlas (3), which was used for gray and white matter segmentation (4–5). Finally, FA, MD, radial diffusivity (RD), and axial diffusivity (AD) were calculated, and statistical analyses were made in 18 bilateral brain areas (6).

The native space masks were eroded using a 2D kernel of 3 × 3 × 1 voxels to avoid partial volume effects and to minimize possible impacts of misregistration. All segmentation outputs were visually inspected and corrected manually if necessary. Besides FA and MD, RD and AD were also calculated (using eigenvalues) and statistical analyses were performed for the following bilateral eroded gray and white matter regions of interest (ROIs): hippocampus, amygdala, neocortex, corpus callosum, corpus callosum genu, anterior commissure, external and internal capsule, inferior colliculus, fornix, fimbria, substantia nigra, hypothalamus, basal ganglia, thalamus, and central gray. We also included the rest of forebrain and midbrain as ROIs. Major steps of diffusion data processing are depicted in Figure 2.

#### MR Volumetry

**Figure 3:**
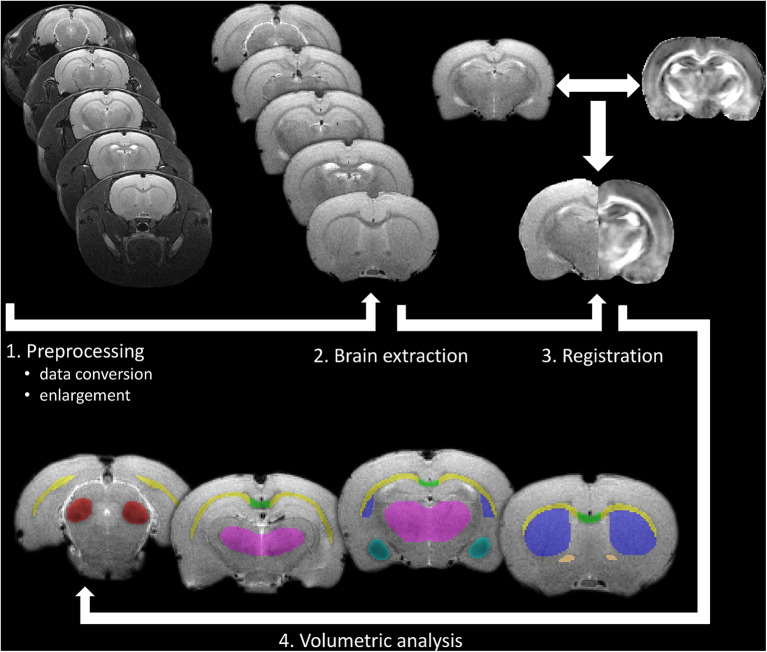
Processing pipeline of the *in vivo* volumetric analysis. After pre-processing of the raw data as a first step, brain extraction was made to improve registration accuracy to a rat brain atlas (2 and 3), which was used for gray and white matter segmentation. Finally, volumetric analysis was performed on manually corrected brain areas using FSLstats (4).

Volumetric analysis was performed in gray and white matter structures, where diffusion abnormalities were found (amygdala, corpus callosum, anterior commissure, external capsule, inferior colliculus, basal ganglia, and thalamus). For MR volumetry, T2-weighted images were spatially registered into a rat brain DTI atlas (Rumple et al., 2013) space using seven degrees-of-freedom linear image registration. Then, the inverse of the transformation from T2 space to atlas space was applied to align the segmented brain masks to T2 space, where volumetric analyses were performed. After that, all segmentation outputs were visually inspected and corrected manually if necessary and the volume of each segmented brain area was calculated by FSLstats, a part of the FSL. Details of MR volumetry can be seen in Figure 3.

### Behavioral Assessment

#### Open Field Test

Locomotor activity was measured using the open field test (OFT) in the *acute* phase of the stress protocol (Week 1, see Figure 1). The OFT arena was a black-colored plywood box with a size of 57.5 × 57.5 cm (length × width) surrounded by 39.5 cm high walls. The floor of the arena was divided with light gray painted lines to four by four equal squares. The four squares in the middle of the arena, which were not bordered by walls, were considered together as the center area of the arena. The rats were allowed to explore the OFT arena for 5 min. After each session, the box was thoroughly cleaned using 20 v/v% ethanol. The sessions were recorded using a high-speed video camera (JVC super LoLux, JVC KENWOOD, Yokohama, Japan), and Ethovision XT10 software (Noldus, Wageningen, Netherlands). During the sessions, the number of line crossings and center area entries of the rats were registered.

#### Novel Object Recognition Test

Recognition memory performance of the animals was tested in the novel object recognition test (NOR) in three occasions: once during the stress procedures and twice during the *recovery* period (Figure 1). The same apparatus (box) was used in the NOR test as in the OFT with the same video tracking system. The NOR test consisted of two trials. In the 1st trial (acquisition), two identical objects were put in the arena, and the rats were allowed to explore them for 3 min. After either 30 min or 3 h retention time, a 2nd trial (recognition) was run with one object kept from the 1st trial (familiar object) and a novel object was introduced, which had never been seen by the animal before. Observation behavior of the animals in the second trial was recorded for 3 min. During the retention period, rats were not transferred back to the animal house but were kept in normal home cages in a dark room located next to the testing room. In both trials, time spent with the exploration of one and the other object was recorded. The animal was considered to explore a given object, when he sniffed the object or put his nose close to it while facing the object.

Three different object pairs were used. The object pairs were distributed randomly between animals and experimental sessions in a counterbalanced latin-square design. In the *acute* stress phase and shortly after the end of the stress protocol (Week 2 and 4, respectively, see Figure 1), the NOR test was run with 30 min retention time between the two trials, while in the stress *recovery* phase (Week 5), 3 h retention time was used.

In the 1st and the 2nd trials of each NOR test session, overall exploratory activity (SumE_1_ and SumE_2_, respectively) was measured by summing the exploration times for the two objects. In the 2nd trial, the time spent with the exploration of the novel (E_*n*_) and the familiar (E_*f*_) objects were compared by calculating a discrimination index (*DI*) using the following equation: DI = (E_*n*_ − *E*_*f*_)/(E_*n*_ + E_*f*_). The DI was a positive number if the novel object was observed longer, while the DI was negative if the familiar object was observed longer, and the DI was around zero if the two objects were observed for an equally long time. Furthermore, a habituation index (HI) was also calculated using the following equation: HI = (SumE_1_/2) − (${\text{E}}_{\text{f}}^{*}$SumE_1_/SumE_2_). The habituation index indicated the extent of decrease in the interest toward the familiar object in the 2nd trial of the NOR test. Only those rats were included in the statistical analysis, who observed both objects and observed them together for at least 5 s in the 2nd trial.

#### Elevated Plus Maze Test

Anxiety-like behavior was tested in the conventional elevated plus maze test (EPM) on the last day of the stress protocol (Week 3, see Figure 1). The EPM apparatus consisted of a central square (11.5 × 11.5 cm) and of four orthogonally situated and equally long arms (45 cm long and 11.5 cm wide) forming a symmetrical plus shape. Two arms had no walls (open arms) and two were enclosed by walls 37 cm in height. The maze stood on an about 100 cm high stand. Rats were placed in the center of the plus maze (i.e., where the four arms met), and they were allowed to explore the maze for 5 min. Time spent in the open arms was recorded during the experiments to assess anxiety-like behavior of the rats.

#### Morris Water Maze Test

Short- and long-term spatial memory of the rats was tested in the Morris water maze apparatus (MWM) 7 weeks after the end of the stress protocol (Week 10 on Figure 1). For the MWM test, we used a blue, circular pool, 180 cm in diameter and 90 cm in height (Ugo Basile, Gemonio, Italy). Four points around the circumference of the pool were designated as North, South, East, and West. On this basis, the area of the pool was divided into four virtual quadrants (NW, SW, SE, and NE). The maze was filled with room-temperature tap water up to the height of 30 cm, and the water was made opaque by mixing 200 g of milk powder and 30 ml of blue food coloring (E131) in it. The rats were trained in the MWM for four consecutive days with one training session per day and four trials per session for each animal on each day. On each trial, a hidden platform was placed in the center of one of the pool quadrants. In each trial, rats were put in the water and were allowed to search for the hidden platform for 120 s. The swimming time elapsed until finding the platform (i.e., sitting on it) was measured as escape latency. If the platform was not found, rats were transferred to the platform and the cutoff time (120 s) was recorded as escape latency. Platform locations were randomly and equally assigned to rats and remained the same for a given rat in every trial on the same day. The quadrant from where the animal started swimming was changed clockwise in the four consecutive trials on a given day. Experiments were recorded using a Basler GenI acA1300 GigE camera (Basler AG, Ahrensburg, Germany). Data were processed onto a computer, where Ethovision X10 software (Noldus, Wageningen, Netherlands) was used for video image recording and data analysis. Short-term learning curves were established by analyzing changes in escape latency from trial to trial on the 1st day of training. Furthermore, escape latency in individual trials were averaged day by day (DayAVG), and long-term learning curves were established by analyzing changes in average escape latency from day to day in the 4-days-long training course.

### Statistical Analysis

Data analyses were performed using IBM SPSS Statistics for Windows, Version 23.0 (IBM Corp., Armonk, NY, USA).

Before using statistical tests, the homogeneity of variance was inspected by Levene's test, while the normality of data was assessed by Shapiro–Wilk statistics. Welch's correction was applied for data demonstrating unequal variances. Prior to the analysis of variance, the data were tested for normality, outliers, and sphericity to ensure the assumptions of repeated measures test. The assumptions of mixed design ANOVA were satisfied, as judged by testing for normality, outliers, homogeneity of variances, and sphericity.

One-way repeated measures ANOVA was performed for each group and structure separately to compare changes in diffusion and volumetric metrics among the time points.

A mixed design ANOVA was used to determine whether any change in MRI metrics is the result of the interaction between the type of group (control vs. stressed) and time. Short-term and long-term learning curves of control and stressed animals were also compared using a mixed design ANOVA, where main effects of TRAINING trials/days (repeated measures) and STRESS (between-subject) and TRAINING × STRESS interaction were tested.

Differences between stressed and control groups were assessed in each time point with independent samples *t*-test (MRI metrics, EPM, DI, and HI in the NOR) or Mann–Whitney *U*-test (OFT). In the NOR test, normal recognition memory performance of a rat was assessed by comparing the difference between the time spent with the novel and with the familiar object using paired *t*-test.

Finally, within-group correlations between MRI metrics and behavioral data were measured separately in the control and the stressed groups using Pearson's or Kendall's tests depending on the normality of the data. Behavioral data were analyzed in pairs with the corresponding (time-matched) MRI data. Thus, results of OFT and NOR-1 experiments were compared with the *acute* phase MRI data, results of EPM and NOR-2 experiments were compared with the *chronic* phase MRI data, and results of NOR-3 and MWM experiments were compared with the *recovery* phase MRI data.

Bonferroni correction was applied for one-way repeated measures and mixed design ANOVA to adjust for multiple comparisons. For one-way repeated measures ANOVA, a level of *p* < 0.002 (*p*′ = 0.05/18 ≈ 0.002) was defined significant in case of DTI metrics, while it was set *p* < 0.007 (*p*′ = 0.05/7 ≈ 0.007) in MR volumetry. Otherwise, results were considered significant at *p* ≤ 0.05.

## Results

### Stress-Induced Changes in White Matter Structures

As expected, the most pronounced stress-induced changes were observed in the white matter. Our MRI measurements revealed that stress significantly altered diffusion properties of the following white matter structures: corpus callosum (CC), external capsule (EC), and anterior commissure (AC).

#### Corpus Callosum

**Figure 4:**
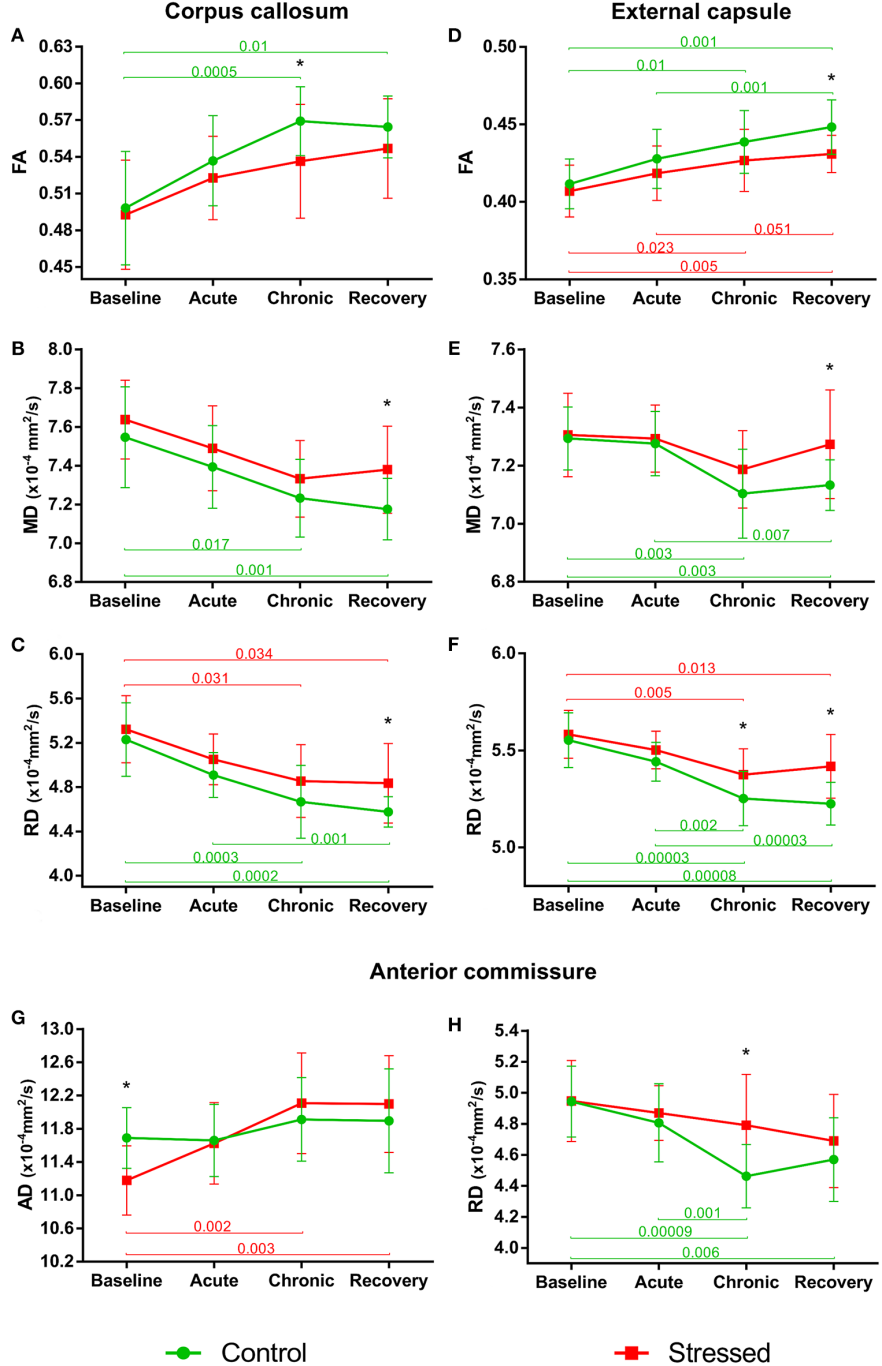
Stress-induced changes of DTI metrics in white matter structures. Longitudinal within- and between-group differences of DTI metrics in the corpus callosum **(A–C)**, external capsule **(D–F)**, and anterior commissure **(G,H)**. Note that most values changed significantly over time as the animals matured. Data are means ± *SD*. Asterisks indicate significant differences between the control and stress groups at the given time point. FA, Fractional anisotropy; MD, Mean diffusivity; RD, Radial diffusivity; AD, Axial diffusivity.

In control animals, the FA values significantly increased over time as the animals matured [*F*_(3, 36)_ = 11.897, *p* = 0.000015], but this developmental change was hindered in the stressed rats [*F*_(3, 36)_ = 4.331, *p* = 0.010] (Figure 4A). MD values decreased significantly over time in controls [*F*_(3, 39)_ = 9.866, *p* = 0.000057], and again this progressive change was not significant in the stressed group [*F*_(3, 36)_ = 5.062, *p* = 0.005] (Figure 4B). RD showed a remarkable decrease over time in both the control [*F*_(3, 39)_ = 18.126, *p* < 0.0000001] and stressed animals [*F*_(3, 36)_ = 7.181, *p* = 0.000672] (Figure 4C), while none of the groups showed time-related AD change. Significant between-group differences were found in FA [*F*_(1, 24)_ = 4.584, *p* = 0.043], MD [*F*_(1, 25)_ = 8.382, *p* = 0.008], and RD values [*F*_(1, 25)_ = 8.555, *p* = 0.007] without any group × time interaction. In the *chronic* stress period, FA values were significantly lower in stressed rats, but no differences were found in the *baseline, acute*, or *recovery* phases (Table 1 and Figure 4A). In the *recovery* period, the MD and RD values were significantly higher in the stressed rats (Table 1 and Figures 4B,C). The volume of CC did not show any change (Table 1).

**Table 1:** Diffusion and volumetric data of white matter structures with between group differences at different time points.

**MRI metrics**	**Brain structure**	**Group (*n*)**	**Baseline**	***p*-value**	**Acute stress**	***p*-value**	**Chronic stress**	***p*-value**	**Recovery**	***p*-value**
FA	CC	Control (13)	0.50 ± 0.05	ns	0.54 ± 0.04	ns	0.57 ± 0.03	0.04^c^	0.56 ± 0.03	ns
		Stress (13)	0.50 ± 0.04		0.52 ± 0.03		0.54 ± 0.05		0.55 ± 0.04	
	EC	Control (14)	0.41 ± 0.02	ns	0.43 ± 0.02	ns	0.44 ± 0.02	ns	0.45 ± 0.02	0.007
		Stress (13)	0.41 ± 0.02		0.42 ± 0.02		0.43 ± 0.02		0.43 ± 0.01	
	AC	Control (14)	0.49 ± 0.03	ns	0.51 ± 0.03	ns	0.55 ± 0.03	ns	0.53 ± 0.03	ns
		Stress (13)	0.48 ± 0.03		0.50 ± 0.03		0.52 ± 0.04		0.53 ± 0.03	
MD^a^	CC	Control (14)	7.55 ± 0.26	ns	7.39 ± 0.21	ns	7.23 ± 0.20	ns	7.18 ± 0.16	0.011
		Stress (13)	7.64 ± 0.20		7.49 ± 0.22		7.33 ± 0.20		7.38 ± 0.22	
	EC	Control (14)	7.29 ± 0.11	ns	7.28 ± 0.11	ns	7.10 ± 0.15	ns	7.13 ± 0.09	0.024^d^
		Stress (13)	7.31 ± 0.14		7.29 ± 0.12		7.19 ± 0.13		7.27 ± 0.19	
	AC	Control (14)	7.16 ± 0.21	ns	7.11 ± 0.22	ns	6.97 ± 0.18	ns	7.02 ± 0.30	ns
		Stress (10)	7.03 ± 0.24		7.09 ± 0.17		7.08 ± 0.08		7.19 ± 0.31	
AD^a^	CC	Control (14)	12.18 ± 0.72	ns	12.36 ± 0.72	ns	12.36 ± 0.54	ns	12.38 ± 0.50	ns
		Stress (13)	12.27 ± 0.74		12.37 ± 0.71		12.29 ± 0.56		12.47 ± 0.47	
	EC	Control (14)	10.77 ± 0.18	ns	10.95 ± 0.30	ns	10.81 ± 0.34	ns	10.95 ± 0.20	ns
		Stress (13)	10.75 ± 0.30		10.88 ± 0.30		10.81 ± 0.32		10.99 ± 0.28	
	AC	Control (13)	11.69 ± 0.37	0.003	11.66 ± 0.43	ns	11.91 ± 0.50	ns	11.90 ± 0.62	ns
		Stress (12)	11.18 ± 0.42		11.63 ± 0.49		12.11 ± 0.61		12.10 ± 0.58	
RD^a^	CC	Control (14)	5.23 ± 0.33	ns	4.91 ± 0.20	ns	4.67 ± 0.33	ns	4.58 ± 0.14	0.019
		Stress (13)	5.32 ± 0.30		5.05 ± 0.23		4.86 ± 0.33		4.84 ± 0.36	
	EC	Control (14)	5.55 ± 0.14	ns	5.44 ± 0.10	ns	5.25 ± 0.14	0.027	5.23 ± 0.11	0.001
		Stress (13)	5.58 ± 0.12		5.50 ± 0.10		5.37 ± 0.13		5.42 ± 0.16	
	AC	Control (14)	4.94 ± 0.23	ns	4.81 ± 0.25	ns	4.46 ± 0.20	0.004	4.57 ± 0.27	ns
		Stress (13)	4.95 ± 0.26		4.87 ± 0.18		4.79 ± 0.33		4.69 ± 0.30	
Volume^b^	CC	Control (14)	5.24 ± 0.59	ns	5.25 ± 0.51	ns	5.42 ± 0.54	ns	5.62 ± 0.42	ns
		Stress (12)	5.18 ± 0.57		5.30 ± 0.65		5.79 ± 0.64		5.79 ± 0.69	
	EC	Control (14)	50.88 ± 5.12	0.032	53.76 ± 4.55	0.032	55.34 ± 6.67	0.062	55.83 ± 5.30	0.032
		Stress (12)	55.46 ± 5.09		58.41 ± 5.90		59.98 ± 5.19		61.30 ± 6.96	
	AC	Control (14)	3.35 ± 0.87	ns	3.07 ± 0.86	ns	3.83 ± 0.95	ns^c^	3.50 ± 0.84	ns
		Stress (11)	3.16 ± 0.84		3.02 ± 0.56		3.66 ± 0.89		3.03 ± 0.49	

*A few subjects were excluded from the analysis when assumptions like no significant outliers or normality were not met*.

*CC, corpus callosum; EC, external capsule; AC, anterior commissure*.

a *Values are expressed in units of × 10^−4^ mm^2^/s*.

b *Values are expressed in units of mm^3^ without scaling*.

c *Welch-corrected p-values*.

d *Without or with marginally significant between-group difference assessed by mixed design ANOVA*.

#### External Capsule

FA values increased significantly in a time-dependent manner both in control [*F*_(3, 39)_ = 13.871, *p* = 0.000003] and stressed [*F*_(3, 36)_ = 8.831, *p* = 0.000161] rats, without a significant group × time interaction, while MD was decreased rapidly in control subjects only [*F*_(3, 39_ = 10.368, *p* = 0.000038] (Figures 4D,E). Here, the between-group difference was significant for FA [*F*_(1, 25)_ = 5.109, *p* = 0.033], but not for MD [*F*_(1, 25)_ = 2.999, *p* = 0.096]. In the *recovery* period, FA was significantly lower, and mean MD was higher in stressed rats (Table 1 and Figures 4D,E). RD showed remarkable decrease over time both in control [*F*_(3, 39)_ = 30.157, *p* < 0.0000001] and in stressed animals [*F*_(3, 36)_ = 9.261, *p* = 0.000113] and we found a significant between-group difference [*F*_(1, 25)_ = 9.127, *p* = 0.006] and a group × time interaction [*F*_(3, 75)_ = 2.996, *p* = 0.036] (Figure 4F). In the *chronic* stress period and in the *recovery* phase, RD was significantly higher in stressed rats compared to controls (Table 1 and Figure 4F). The volume of the EC increased significantly as the animals matured both in control [*F*_(3, 39)_ = 11.405, *p* = 0.000017] and in stressed [*F*_(3, 33)_ = 15.108, *p* = 0.000002] rats, and there was also a between-group difference [*F*_(1, 24)_ = 5.477, *p* = 0.028] without group × time interaction probably due to the initial difference in the *baseline* measurements (*p* = 0.032). This difference remained constant over the time (Table 1).

#### Anterior Commissure

AD was significantly increased in the stressed rats [*F*_(3, 33)_ = 12.085, *p* = 0.000017] (Figure 4G), but remained constant in the control group. We found a group × time interaction [*F*_(3, 69)_ = 3.396, *p* = 0.023] without a between-group difference. In the *baseline* period, AD was significantly higher in control rats compared to stressed animals, which most likely contributed to the group × time interaction (Table 1 and Figure 4G). RD significantly decreased in controls [*F*_(3, 39)_ = 13.655, *p* = 0.000003], but not in stressed rats (Figure 4H) and we found a significant between-group difference [*F*_(1, 25)_ = 4.800, *p* = 0.038] as well. In the *chronic* phase, the RD was significantly higher in stressed rats, while no differences were found in the *baseline, acute*, or *recovery* phases (Table 1 and Figure 4H). The volume of AC did not change.

### Stress-Induced Changes in Gray Matter Structures

We could observe stress-induced microstructural alterations in a few gray matter areas as follows: amygdala, inferior colliculus (IC), thalamus, and the basal ganglia (BG).

#### Amygdala

**Figure 5:**
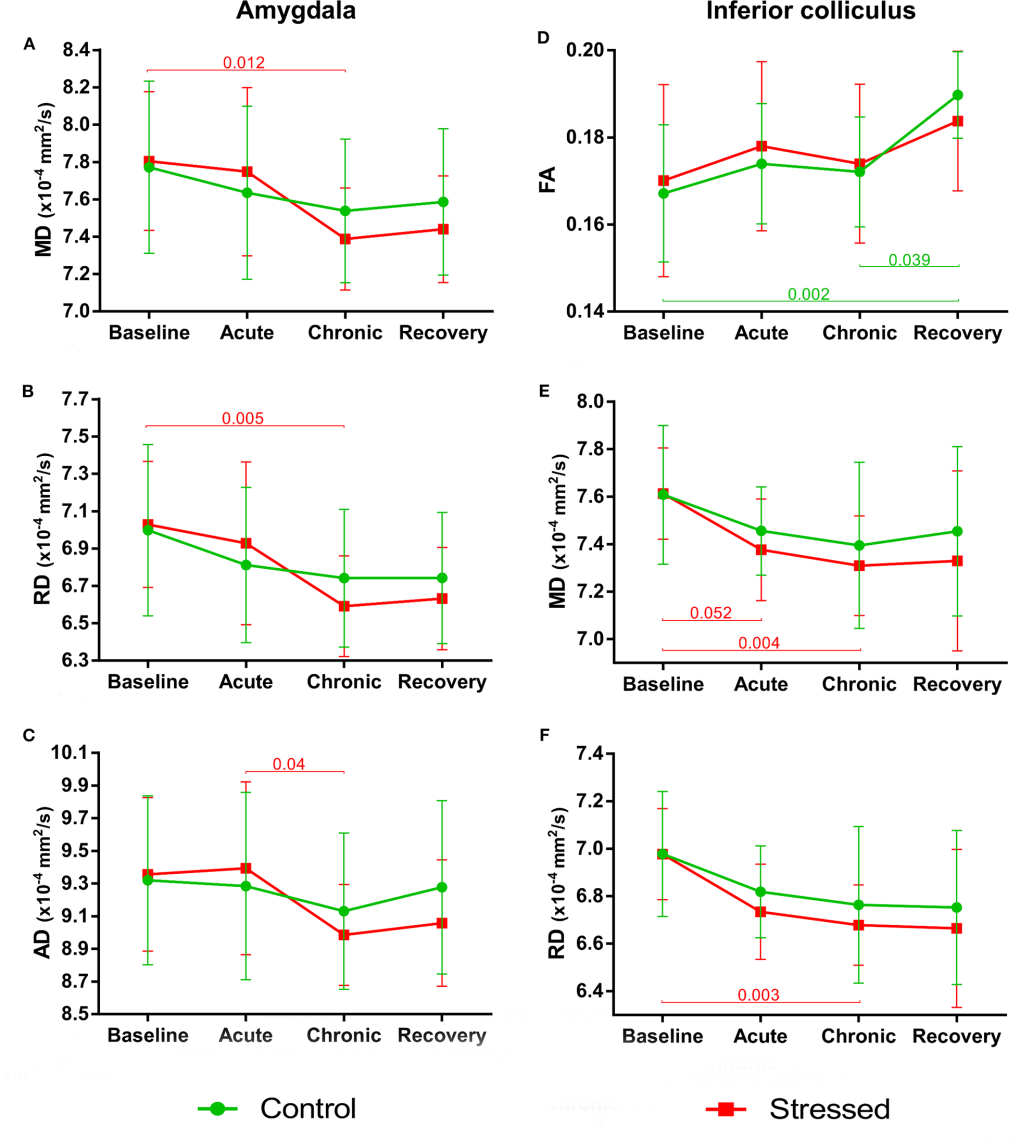
Stress-induced changes of DTI metrics in gray matter areas I. Longitudinal within-group differences of DTI metrics in the amygdala **(A–C)** and inferior colliculus **(D–F)**. Note that most values changed significantly as the animals matured. Data are means ± *SD*. FA, Fractional anisotropy; MD, Mean diffusivity; RD, Radial diffusivity; AD, Axial diffusivity.

Stress reduced MD and RD of the amygdala [*F*_(3, 30)_ = 6.076, *p* = 0.002 and *F*_(3, 30)_ = 5.999, *p* = 0.002, respectively], but these values remained constant in control rats. We found no between-group differences or group × time interaction (Figures 5A,B). The stress-induced decrease of AD was close to the level of significance [*F*_(3, 30)_ = 4.703, *p* = 0.008], while this value did not change in the controls (Figure 5C). We found no between-group differences or group × time interaction. Neither stress nor time affected FA or the volume of the amygdala (see data in the Supplementary Table S1).

#### Inferior Colliculus

As the animals developed, the FA values significantly increased in the control rats [*F*_(3, 39)_ = 7.005, *p* = 0.0007], but did not change in the stress group (Figure 5D). We found no between-group differences or group × time interaction. MD and RD values showed a nearly significant decrease in the stressed rats [*F*_(1.52, 18.25)_ = 6.833, *p* = 0.010 and *F*_(1.41, 16.86)_ = 7.376, *p* = 0.009 both with Greenhouse–Geisser correction], while no change was found in the controls (Figures 5E,F). None of the groups showed significant time-related volume change in the IC; however, there was a between-group difference [*F*_(1, 24)_ = 5.866, *p* = 0.023] without group × time interaction probably due to the initial difference at the *baseline* measurements (*p* = 0.003) (Supplementary Table S1).

#### Thalamus

**Figure 6:**
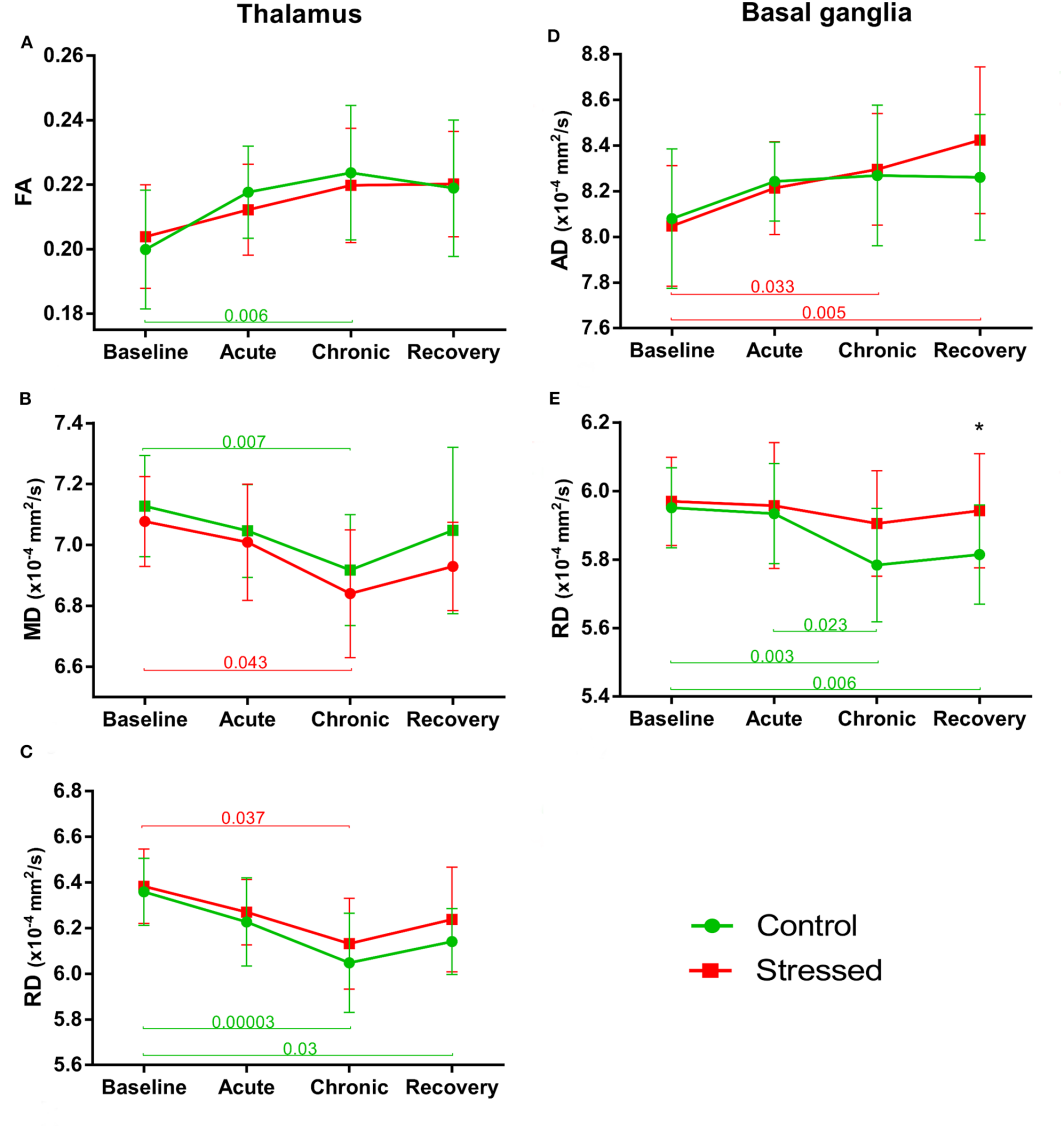
Stress-induced changes of DTI metrics in gray matter areas II. Longitudinal within- and between-group differences of DTI metrics in the thalamus **(A–C)** and basal ganglia **(D,E)**. Note that most values changed significantly over time as the animals matured. Data are means ± *SD*. Asterisk shows significant between-subject differences per time point. FA, Fractional anisotropy; MD, Mean diffusivity; RD, Radial diffusivity; AD, Axial diffusivity.

FA values tended to increase both in the control [*F*_(3, 39)_ = 4.198, *p* = 0.011] and in the stress group over time [*F*_(3, 36)_ = 3.295, *p* = 0.031], but these changes could only approach the level of significance (Figure 6A). A similar effect was found in MD, where a nearly significant decrease was observed in both the stressed [*F*_(3, 36)_ = 3.321, *p* = 0.030] and the control animals [*F*_(3, 39)_ = 4.504, *p* = 0.008] (Figure 6B). AD of thalamus was not altered in any way, but RD decreased significantly in control rats [*F*_(3, 39)_ = 7.782, *p* = 0.0003] over time, while it was only close to the level of significance in the stressed animals [*F*_(3, 36)_ = 4.575, *p* = 0.008] (Figure 6C). The volume of the thalamus did not change and none of the MRI metrics showed any group × time interactions or between group differences (see data in Supplementary Table S1).

#### Basal Ganglia

FA values gradually increased in both the stressed [*F*_(3, 36)_ = 7.852, *p* = 0.00037] and control [*F*_(3, 39)_ = 7.292, *p* = 0.000537] animals without any significant group × time interaction or between-group difference (Supplementary Table S1). MD did not change over time either in the control or in the stressed rats. AD was increased significantly in the stress group [*F*_(3, 36)_ = 7.866, *p* = 0.00036], but did not change in the controls (Figure 6D). Here, there was no significant group × time interaction and between-group difference. In contrast to that, RD significantly decreased in controls [*F*_(3, 39)_ = 7.292, *p* = 0.000537], but did not change over time in the stressed rats (Figure 6E). For RD values, we found nearly significant between-group difference [*F*_(1, 25)_ = 3.339, *p* = 0.080] without group × time interaction. In the *recovery* phase, the RD was higher in the stressed rats compared to the controls, while no alterations were found in the *baseline, acute*, or *chronic* phases (Supplementary Table S1 and Figure 6E). The volume of BG showed significant time-dependent increase in both the stressed [*F*_(1, 99, 19, 87)_ = 7.665, *p* = 0.003 with Greenhouse–Geisser correction and control rats [*F*_(3, 39)_ = 4.898, *p* = 0.0055] without group × time interaction and between-group differences (Supplementary Table S1).

### Behavioral Assessment

Stress exposure had no influence on the cognitive performance, anxiety-like behavior, or locomotor activity of the animals during the *acute* and *chronic* stress periods. We performed an OFT and a NOR test at the end of the 1st week of the stress protocol (*acute stress* period, Figure 1), and an EPM test in the last day of the chronic stress procedures (*chronic stress* period, Figure 1). None of these tests yielded any difference between the control and stressed rats (for details, please see the Supplementary Material).

Stress-induced behavioral differences emerged only in the *recovery* period (Figure 1). In the NOR-3 test, we applied longer retention times—making the task more difficult for the animals—which probably contributed to the higher sensitivity for detecting a cognitive impairment in the stressed rats. In Week 5, stressed animals (*n* = 15) showed no discrimination behavior in the NOR-3 test after a 3-h retention period (observation of novel vs. familiar object: 9.8 ± 1.3 vs. 6.6 ± 0.9, *t* = 1.895, *p* = 0.078), while control animals (*n* = 16) spent significantly more time with the exploration of the novel object (10.5 ± 1.0 vs. 6.5 ± 0.5 s, *t* = 3.270, *p* = 0.006).

A similar difference was present between the control and stressed rats in the MWM, which was done 7 weeks after the end of the stress procedures. Stressed rats showed impaired short-term memory performance in the MWM as the mixed ANOVA indicated a significant main effect of STRESS on the escape latency on the 1st day of the training [*F*_(1, 30)_ = 10.144, *p* = 0.003; *n* = 16 for both groups]. Stressed animals could also learn the location of the platform during the 4 days of training; however, their escape latency was longer during the whole training procedure [main effect of STRESS: *F*_(1, 30)_ = 4.945, *p* = 0.034].

### Correlation Analysis Between the MRI Data and Behavioral Performance

While stress resulted only in mild cognitive impairments, we found that numerous parameters of the cognitive performance showed significant correlations with the DTI and volumetric data (see Tables 2–**4**).

**Table 2:** Correlations between MRI metrics and cognitive performance in the *acute* stress period.

**Brain area**	**NOR-1** **results**	**MRI** **metrics**	**Control**	**Stress**
Amygdala	DI	Vol	*R* = 0.303	*R* = 0.752^*^
	HI	Vol	*R* = 0.339	*R* = 0.757^*^
Anterior commissure	DI	Vol	*R* = 0.667^**^	*R* = 0.244
	HI	Vol	*R* = 0.635^*^	*R* = 0.097
Corpus callosum	DI	MD	*R* = −0.259	*R* = 0.750^*^
	HI	MD	*R* = −0.173	*R* = 0.688^*^
Inferior colliculus	DI	AD	*R* = 0.270	*R* = 0.711^*^
	DI	MD	*R* = 0.306	*R* = 0.689^*^
	HI	MD	*R* = 0.225	*R* = 0.743^*^
	HI	AD	*R* = 0.213	*R* = 0.785^**^
	HI	RD	*R* = 0.212	*R* = 0.636^*^

NOR-1, novel object recognition test performed on the 10th day of the stress exposure; DI, discrimination index; HI, habituation index; AD, axial diffusivity; MD, mean diffusivity; RD, radial diffusivity; Vol, volume; R, Pearson's correlation coefficient;

* p < 0.05;

** *p < 0.01*.

#### Acute Stress Period

We found significant correlations between specific parameters of the MRI data and results of the NOR-1 and OFT. DI and HI of the NOR-1 test correlated with the volume of the amygdala in the stressed rats and with the volume of the AC in the control animals (Table 2). DI and HI values of the NOR-1 test correlated with MD in the CC of the stressed rats and with AD, MD, and RD values of the IC in the stressed rats (Table 2).

We also found significant correlations between specific parameters of the DTI data and results of the OFT. Entries to the center area correlated with FA (τ = 0.486, *p* < 0.05) and RD (τ = −0.509, *p* < 0.05) values of the AC in the control rats.

#### Chronic Stress Period

We found significant correlations between specific parameters of the MRI data and results of the NOR-2 and EPM tests. DI of the NOR-2 test correlated with the volume of the amygdala in the stressed rats (Table 3). In the AC, FA and RD values correlated with the HI of the NOR-2 test of the stressed animals (Table 3). Furthermore, DI and HI values of the NOR-2 test correlated with AD, FA, and MD values in the IC of the stressed rats (Table 3).

**Table 3:** Correlations between MRI metrics and cognitive performance in the *chronic* stress period.

**Brain area**	**NOR-2** **results**	**MRI** **metrics**	**Control**	**Stress**
Amygdala	DI	Vol	*R* = −0.186	*R* = 0.665^*^
Anterior commissure	HI	FA	*R* = 0.079	*R* = −0.614^*^
	HI	RD	*R* = 0.063	*R* = 0.603^*^
Inferior colliculus	DI	FA	*R* = −0.172	*R* = 0.666^*^
	DI	MD	*R* = 0.028	*R* = 0.669^*^
	DI	AD	*R* = −0.024	*R* = 0.733^*^
	HI	AD	*R* = 0.160	*R* = 0.649^*^

NOR-2, novel object recognition test performed few days after the end of the chronic stress exposure; DI, discrimination index; HI, habituation index; AD, axial diffusivity; FA, fractional anisotropy; MD, mean diffusivity; RD, radial diffusivity; Vol, volume; R, Pearson's correlation coefficient;

* *p < 0.05*.

Significant correlations between DTI data and behavior were found also in the EPM. In control rats, FA values of the AC correlated with the time spent in the open arms of the EPM (*R* = −0.539, *p* < 0.05).

#### Recovery Period

Surprisingly, most of the correlations between behavior and MRI data were found in the *recovery* period (Table 4). In this phase, we did a NOR test 2 weeks after the end of the stress (NOR-3) and a MWM test 7 weeks after the end of the stress (Figure 1). The HI of the NOR-3 test correlated with the FA value of the AC in stressed rats and with the MD value of the BG in the control animals (Table 4). Most of the correlations were found between the animal's performance in the MWM and between the MRI data (for details, see Table 4). In the amygdala, RD and MD values correlated with escape latencies of the control (RD, τ = 0.429, *p* < 0.05) and stressed (MD, τ = −0.474, *p* < 0.05; RD, τ = −0.500, *p* < 0.05) rats. In the anterior commissure, the AD, FA, and MD values correlated with the cognitive performance of the control rats and with the AD value of the stressed rats (Table 4). The volume of the BG in the stressed animals correlated with escape latencies, whereas in controls, the FA value correlated with the escape latency (Table 4). In the corpus callosum, the AD and FA values correlated with escape latencies of the control rats and AD and MD values correlated with escape latencies of the stressed rats (Table 4). In the EC, the volume and AD of the stressed rats correlated with escape latency, whereas in the controls, the MD correlated with escape latency (Table 4). In the inferior colliculus, FA values correlated with escape latencies of both the control and stressed rats (Table 4). In the thalamus, the volume as well as AD and RD values of the stressed rats correlated with their escape latencies (Table 4).

**Table 4:** Correlations between MRI metrics and cognitive performance in the *recovery* period.

**Brain area**	**Behavioral task**	**Behavioral parameter**	**MRI metrics**	**Control**	**Stress**
Amygdala	MWM	Day2 escape latency	MD	τ = 0.322	τ = −0.474^*^
	MWM	Day2 escape latency	RD	τ = 0.429^*^	τ = −0.500^*^
Anterior commissure	NOR-3	HI	FA	τ = 0.103	τ = 0.487^*^
	MWM	Trial2 escape latency	FA	τ = −0.501^*^	τ = −0.118
	MWM	Trial3 escape latency	MD	τ = 0.478^*^	τ = −0.295
	MWM	Trial3 escape latency	AD	τ = 0.454^*^	τ = −0.113
	MWM	Day1 escape latency	AD	τ = 0.055	τ = −0.460^*^
Basal ganglia	NOR-3	HI	MD	τ = 0.431^*^	τ = −0.051
	MWM	Trial4 escape latency	Vol	τ = −0.035	τ = −0.510^*^
	MWM	Day1 escape latency	Vol	τ = 0.033	τ = −0.557^*^
	MWM	Day4 escape latency	Vol	τ = 0.331	τ = −0.485^*^
	MWM	Day4 escape latency	FA	τ = 0.486^*^	τ = 0.103
Corpus callosum	MWM	Trial2 escape latency	FA	τ = −0.426^*^	τ = −0.024
	MWM	Trial3 escape latency	FA	τ = −0.408^*^	τ = 0.113
	MWM	Trial3 escape latency	AD	τ = −0.478^*^	τ = −0.033
	MWM	Day3 escape latency	MD	τ = 0.187	τ = −0.458^*^
	MWM	Day3 escape latency	AD	τ = 0.275	τ = −0.452^*^
	MWM	Day4 escape latency	AD	τ = −0.022	τ = −0.555^**^
External capsule	MWM	Day1 escape latency	Vol	τ = −0.143	τ = −0.522^*^
	MWM	Day1 escape latency	AD	τ = −0.253	τ = −0.489^*^
	MWM	Day3 escape latency	MD	τ = 0.416^*^	τ = −0.231
Inferior colliculus	MWM	Trial4 escape latency	FA	τ = 0.046	τ = 0.496^*^
	MWM	Day1 escape latency	FA	τ = 0.033	τ = 0.633^**^
	MWM	Day4 escape latency	FA	τ = −0.398^*^	τ = −0.077
Thalamus	MWM	Day1 escape latency	RD	τ = 0.033	τ = −0.449^*^
	MWM	Day2 escape latency	Vol	τ = −0.099	τ = −0.688^**^
	MWM	Day4 escape latency	AD	τ = 0.000	τ = −0.436^*^

*NOR-3, novel object recognition test which was performed on the 2nd week of the recovery period; MWM, Morris water maze test, which was done on the 7th week of the recovery period; HI: habituation index; AD, axial diffusivity; FA, fractional anisotropy; MD, mean diffusivity; RD, radial diffusivity; Vol, volume; R, Pearson's correlation coefficient; τ, Kendall's tau coefficient*.

* p < 0.05;

** *p < 0.01*.

## Discussion

### Main Findings

Here, we report that chronic stress exposure results in pronounced microstructural changes in white matter structures of rats such as the corpus callosum, anterior commissure, and external capsule. We observed modest microstructural alterations also in gray matter structures such as the amygdala, thalamus, inferior colliculus, and basal ganglia. These stress-induced microstructural differences developed gradually, and many of them were lasting and remained even after the end of the stress exposure. Since we used young adult rats, consequently most of the DTI parameters changed as the rats matured. In many cases, stress exposure hindered these developmental changes of DTI values. We found no effect of repeated restraint stress on the volume of the examined brain areas.

In the present experiment, the chronic stress exposure induced only modest cognitive impairments, but these changes were long-lasting, i.e., the cognitive performance was impaired even several weeks after the end of the stress protocol (in the *recovery* period). Notably, we found numerous correlations between the cognitive performance of the rats and the DTI metrics of the amygdala, anterior commissure, basal ganglia, corpus callosum, external capsule, inferior colliculus, and thalamus. To the best of our knowledge, we are the first to document correlations between cognitive capacities and stress-induced microstructural changes detected by DTI. Overall, these data extend and complement the earlier histopathological findings documenting multifaceted cellular alterations in the brains of chronically stressed animals. Importantly, our present data on the stress-induced reduction of FA and increased MD and RD values of white matter structures are in good harmony with the findings of recent meta-analyses revealing similar microstructural changes in stress-related psychiatric disorders (Kelly et al., 2018; Dennis et al., 2019; Favre et al., 2019; Koshiyama et al., 2019; van Velzen et al., 2019).

### Stress-Induced Structural Changes Detected by *in vivo* DTI Measurements

A large body of evidence document stress-induced cellular alterations of neurons and glia in the brains of humans and animals (see, e.g., Lucassen et al., 2014; Lupien et al., 2018). Most of these data stem from postmortem investigations, but a growing number of neuroimaging data complement these findings. *In vivo* imaging experiments of chronically stressed animals reveals significant changes in large-scale functional connectivity networks (Henckens et al., 2015; Gass et al., 2016; Magalhaes et al., 2018, 2019), while proton MR spectroscopy documents altered brain metabolites and neurotransmitter levels (Czéh et al., 2001; Khan et al., 2018; Magalhaes et al., 2019). However, these findings are not without controversies. For example, some studies reported on stress-induced volume reductions of specific brain structures (Li et al., 2017; Magalhaes et al., 2018), whereas other experiments could not substantiate that (Henckens et al., 2015, and our present data). The available diffusion MRI findings are also ambiguous. For example, a recent study using tract-based spatial statistical analysis approach reported that stress can increase FA and reduce MD and RD in several white matter bundles after 2 weeks of repeated inescapable stress (Magalhaes et al., 2017). Another study also reported increased FA in the hypothalamus and hippocampal CA3 in stress-susceptible mice, which were subjected to 10 days of social defeat stress (Anacker et al., 2016). Yet, another research group found no evidence for white matter microstructural changes in rats exposed to 10 days of repeated immobilization stress (Henckens et al., 2015). A study investigating a genetic rat model of depression documented decreased FA in the CC and AC and increased MD in the CC (Zalsman et al., 2017). In our present experiment, we also found reduced FA and increased MD and RD values in several white matter structures indicating myelin destruction (Alexander et al., 2007). However, the between-group differences and the group × time interactions of DTI metrics were different in the various brain areas. Most likely, this was due to the fact that DTI metrics have a complex origin and show a regionally varying, non-linear change across a wide age range (Lebel et al., 2012; Mengler et al., 2014; Kulikova et al., 2015). In general, FA increases, while MD and RD decrease as white matter structures mature, but the temporal dynamics are inhomogeneous in the different pathways (Lebel et al., 2012; Kulikova et al., 2015), which could explain the variances in our data. Despite all that, our data suggest that the stress-induced microstructural changes are long-lasting and may remain detectable even weeks after the end of the stress procedures. This finding was supported by a recent study, which investigated the lasting effects of stress and found that it may take up to 8 weeks of recovery to normalize the stress-induced microstructural changes (Khan et al., 2018).

In our present experiment, the rats were 8 weeks old when we started to stress them, which means that they were at the age of late adolescence or young adulthood as defined by Tirelli and co-workers (Tirelli et al., 2003). At this age, the rat brain is almost completely matured. After the end of the 2nd month, brain areas do not change in their volume anymore, but there are still significant changes in myelination between the 2nd and 3rd months, which are also reflected by the significant reductions of MD and RD values of the whole brain (Mengler et al., 2014). There are significant regional differences, e.g., the neocortex appears to be fully matured by the age of 2 months, but there are still significant changes in neuron numbers of the striatum between the 2nd and 3rd months (Mengler et al., 2014). There is postmortem histological evidence that maturation of the axon fibers lasts even longer and myelination is still changing up to 6 months of age in rats (Mengler et al., 2014).

It is well-documented that during adolescence, individuals (both humans and experimental animals) are more susceptible to stress because during this age, stress exposure leads to more prolonged glucocorticoid exposure and appears to alter the development of emotional and cognitive systems, which may result in enduring mild deficits in learning and memory tasks or increased anxiety-like behavior (Spear, 2000; McCormick et al., 2010, 2017; Romeo et al., 2016). In the human brain, FA values continuously increase during childhood and adolescence and reach a peak between 20 and 42 years of age, while MD shows an opposite trend, decreasing first, reaching a minimum at 18–41 years, and then increasing later in life (Lebel et al., 2012). Numerous human DTI studies documented white matter abnormalities in individuals who experienced adverse life events during their childhood and adolescence. Specifically, microstructural white matter changes were found in adolescents exposed to childhood maltreatment (Huang et al., 2012) or in young adults who witnessed domestic violence (Choi et al., 2012) or experienced parental verbal abuse (Choi et al., 2009) during their early age. Childhood maltreatment can result in reduced corpus callosum area (Teicher et al., 2004) and reduced FA values in the corpus callosum (Jackowski et al., 2008). Notably, a recent study that compared the timing of the adverse experiences found that stress seems to induce different types of changes in DTI metrics depending on the age when individuals experienced the stress (Jensen et al., 2018).

To the best of our current knowledge, the microstructural alterations and anisotropy of gray and white matter structures are the consequences of altered myelination, fiber density, neuronal morphology, and synaptogenesis (Evans, 2013; Jensen et al., 2018). There are very few studies that analyzed the stress-induced structural changes with both neuroimaging and conventional light microscopy techniques (but see Khan et al., 2019). At the same time, plenty of postmortem studies document stress-induced reductions of dendritic complexity of pyramidal cells in the hippocampus (Magarinos et al., 1996) and neocortex (Cook and Wellman, 2004; Radley et al., 2004), and reduced synapse numbers (Sandi et al., 2003; Hajszan et al., 2009; Maras et al., 2014; Csabai et al., 2018). Furthermore, stress affects not only neurons but also the level of myelination and oligodendrocytes (Czéh et al., 2007; Miyata et al., 2016; Yang et al., 2016; Lehmann et al., 2017; Liu J. et al., 2018) and can reduce fiber density as well (Kitayama et al., 1997; Csabai et al., 2018).

Overall, these *in vivo* MR measurements may help us develop objective biomarkers (Kelly et al., 2018; Koshiyama et al., 2019; Nugent et al., 2019; van Velzen et al., 2019) and predict treatment response in mental disorders (Crossley et al., 2017; Coenen et al., 2019; Davis et al., 2019).

### Correlation Between the DTI Parameters and Cognitive Behavior

Several studies aimed to find correlations between stress-induced behavioral changes and MRI findings. Most of these studies analyzed the correlation between stress susceptibility and neuroimaging data. For example, Anacker et al. investigated the neurobiological mechanisms underlying stress susceptibility using structural MRI and DTI to determine neuroanatomic differences between stress-susceptible and stress-resilient mice (Anacker et al., 2016). They scanned the brains *ex vivo* and found that social avoidance correlated negatively with local volume of the cingulate cortex, nucleus accumbens, thalamus, raphe nuclei, and bed nucleus of the stria terminalis (Anacker et al., 2016). Furthermore, they found a positive correlation between social avoidance and the volume of the ventral tegmental area, habenula, periaqueductal gray, cerebellum, hypothalamus, and hippocampal CA3 (Anacker et al., 2016). They also observed increased FA in the hypothalamus and hippocampal CA3 and different structural covariance between brain regions in susceptible and resilient mice (Anacker et al., 2016). Others reported on a significantly lower FA in the right ventral hippocampus of the stress-susceptible mice prior to the chronic stress exposure, suggesting that pre-existing microstructural abnormalities may result in stress susceptibility (Liu X. et al., 2018). Another study documented substantial increase (45%) in the volume of the amygdala of a rat strain that is susceptible to the repeated stress procedure (F344 rats; Bourgin et al., 2015). More recently, Bonnefil et al. documented that region-specific myelin differences define the behavioral consequences of the chronic social defeat stress in mice (Bonnefil et al., 2019).

In our present study, we expected that stress would result in pronounced cognitive deficits and elevated anxiety-like behavior in the stressed animals. In contrast, we observed only mild cognitive impairments in the stressed rats, but we found numerous correlations between the DTI parameters and the cognitive performance of the animals. The most pronounced correlations were found between the DTI values of the amygdala, IC, AC, CC, EC, and between the behavioral performance in the NOR test. Surprisingly, in the present experiment, the MRI data recorded 2 weeks after the end of the stress correlated best with escape latencies in the MWM test, which was done 5 weeks after the MRI scans. Numerous reports document that learning can induce changes in structural neuroplasticity, which can be then measured with DTI (e.g., Blumenfeld-Katzir et al., 2011; Zatorre et al., 2012; Ding et al., 2013; Nasrallah et al., 2016; Hofstetter and Assaf, 2017; Huber et al., 2018). These studies suggest neuronal activity-dependent changes in myelination levels and the remodeling of the existing myelin sheath as an underlying cellular process behind the altered DTI metrics (Fields, 2015; Kaller et al., 2017). Our present data extend these findings and document that the stress-induced microstructural changes can also predict the alterations of cognitive performance.

### Clinical Relevance

Stress is a major contributing factor to the development of various stress-related psychiatric disorders such as post-traumatic stress disorder (PTSD), depressive disorders, or schizophrenia. Since these disorders are common and impose severe socio-economic burden, substantial efforts have been devoted to mimic these conditions in experimental animals. Chronic stress models are typically regarded as valid models for major depressive disorder (MDD) (see, e.g., Willner, 1997; Czéh et al., 2016), and the translational value of neuroimaging has been well-demonstrated in earlier studies using chronic psychosocial stress protocols, which had led to alterations in brain volume, functional connectivity, and metabolite levels comparable to human depression (Czéh et al., 2001; Grandjean et al., 2016). At the same time, numerous clinical studies seek for neuroimaging biomarkers, which could help the objective diagnosis of stress-related psychiatric disorders. The ENIGMA Schizophrenia DTI Working Group analyzed the data of 2,359 healthy controls and 1,963 schizophrenia patients and found significant reductions of FA in schizophrenia patients, especially in the corpus callosum, but also in 20 out of 25 major white matter fasciculi (Kelly et al., 2018). They also found significantly higher MD and RD in the schizophrenia group (Kelly et al., 2018). These data have been confirmed more recently by another mega-analysis comparing white matter microstructural differences between healthy controls and sufferers of schizophrenia, bipolar disorder, autism spectrum disorder, and MDD (Koshiyama et al., 2019). These comparisons document significantly lower FA and higher MD and AD values for most of the major white matter bundles in these disorders (Koshiyama et al., 2019). Another study, the ENIGMA MDD working group, examined white matter anisotropy and diffusivity in 1,305 MDD patients and 1,602 healthy controls and found subtle, but widespread alterations showing reduced FA in adult MDD patients compared to controls in 16 out of 25 white matter tracts (van Velzen et al., 2019). The largest difference was depicted in the corpus callosum and widespread higher RD values were also observed (van Velzen et al., 2019). Notably, in our present study, we also found lower FA and higher MD and RD values in the CC and EC of the stressed rats, which is in good harmony with these clinical findings. Similar data are available on PTSD patients: significantly lower FA values of the CC have been documented in traumatized individuals (Hu et al., 2016; O'Doherty et al., 2018; Siehl et al., 2018; Dennis et al., 2019), in maltreated children (Jackowski et al., 2008), or in adolescents after childhood sexual abuse (Rinne-Albers et al., 2016).

## Conclusions

In conclusion, our data provide further support to the translational value of DTI studies and suggest that chronic stress exposure can result in similar white matter microstructural alterations, which have been documented in stress-related psychiatric disorders. Furthermore, we report here significant correlations between cognitive performance and stress-induced microstructural changes detected by DTI. Overall, the *in vivo* imaging findings complement the earlier postmortem histopathological data and suggest structural disconnectivity in stress-related pathologies.

## Data Availability Statement

All datasets generated for this study are included in the article/Supplementary Material.

## Ethics Statement

The animal study was reviewed and approved by the Hungarian Ethical Committee on Animal Research according to the Ethical Codex of Animal Experiments (License No. BA02/2000-12/2015).

## Author Contributions

BC and SN had the concept, designed the experiments, and drafted the manuscript. ZV and DC did the stress procedures. NB, ZBa, and IH did all the behavioral testing and analyzed the behavioral data. AV, SN, and GP did the MRI measurements and analyzed the MRI data. AM, TD, and ZBe provided supervision for the experimental procedures and contributed to the interpretation of the data. All authors contributed to the writing of the paper and revised it critically for important intellectual content and approved the final version to be published and agreed to be accountable for all aspects of the work in ensuring that questions related to the accuracy or integrity of any part of the work are appropriately investigated and resolved.

## Conflict of Interest

The authors declare that the research was conducted in the absence of any commercial or financial relationships that could be construed as a potential conflict of interest.

## Abbreviations

AC: anterior commissure
AD: axial diffusivity
BG: basal ganglia
CC: corpus callosum
DI: discrimination index
DTI: diffusion tensor imaging
EC: external capsule
EPM: elevated plus maze test
FA: fractional anisotropy
HI: habituation index
IC: inferior colliculus
MD: mean diffusivity
MDD: major depressive disorder
MR: magnetic resonance
MRI: magnetic resonance imaging
MWM: Morris water maze
NOR: novel object recognition test
OFT: open field test
RD: radial diffusivity
ROI: regions of interest.
