# Supplementary material for: Stress-Induced Microstructural Alterations Correlate With the Cognitive Performance of Rats: A Longitudinal in vivo Diffusion Tensor Imaging Study
Source: Front Neurosci. 2020 Jun 3;14:474. doi: 10.3389/fnins.2020.00474 (PMC7283577; doi:10.3389/fnins.2020.00474)
Supplement: Supplementary file 1 [file Table_1.DOCX]

Supplementary Material

Stress-induced microstructural alterations correlate with the cognitive performance of rats: A longitudinal in vivo diffusion tensor imaging study.

**Szilvia Anett Nagy** §**, Anett Vranesics** §**, Zsófia Varga, Dávid Csabai, Nóra Bruszt, Zsolt Kristóf Bali, Gábor Perlaki, István Hernádi, Zoltán Berente, Attila Miseta, Tamás Dóczi, Boldizsár Czéh***

§ These authors contributed equally

*** Correspondence:**

Boldizsár Czéh, MD, PhD, DSc, **E-mail address:** [czeh.boldizsar@pte.hu](mailto:czeh.boldizsar@pte.hu)

# Supplementary Data

## Behavioral assessment

We did an open field test in the acute phase of the stress protocol (Figure 1) to examine the locomotor activity of control and stressed rats (*n*=16 for both groups). The results of OFT did not yield any difference in the number of crossings (Control vs. Stressed: 82.5±6.53 vs. 85.25±6.93; U=129.5, p=0.956) and in the number of inner area entries (5.88±1.00 vs. 4.75±0.69; U=119.5, p=0.752) between the two groups.

In the acute stress period, the cognitive performance of the animals also did not differ in the NOR-1 test (Control: *n*=15 and Stressed: *n*=13): the DI and HI values were similar in the acute phase (Week 1: DI: 0.118±0.050 vs. 0.174±0.120, t=−0.831, df=19.5, p=0.431; HI: 1.615±0.760 vs. 2.788±1.59, t=−0.634, df=21.3, p=0.533). Similarly, in the chronic stress period, we could not see any difference between the animals (NOR-2 in Week 4: *n*=16 control and *n*=14 stressed rats; DI: 0.236±0.060 vs. 0.253±0.872, t=−0.162, df=28, p=0.872; HI: 2.310±0.590 vs. 3.185±1.310, t=−0.611, df=18.1, p=0.549). Both control and stressed animals observed the novel object significantly longer than the familiar object in Week 1 (p=0.014 for the Control and p=0.028 for Stressed groups) and in Week 4 (p=0.001 and p<0.001 for Control and Stressed groups, respectively).

The EPM test that was performed on the last day of the immobilization stress (Figure 1), but we could not detect any difference in the anxiety-like behavior of the control and stressed animals (time spent on the open arms: 97.0±9.3 s vs. 107.5±10.3 s, *n*=16 for both groups).

# Supplementary Table

| **Table 1. Diffusion and volumetric data of grey matter structures with between group differences at different time points.** | | | | | | | | | | |
| --- | --- | --- | --- | --- | --- | --- | --- | --- | --- | --- |
| **MRI metrics** | **Brain structure** | **Group (n)** | **Baseline** | **p-value** | **Acute stress** | **p-value** | **Chronic stress** | **p-value** | **Recovery** | **p-value** |
| FA | Amy | Control (n=14) | 0.20±0.021 | ns | 0.21±0.01 | ns | 0.20±0.02 | ns | 0.21±0.02 | ns |
|  |  | Stress (n=13) | 0.20±0.01 |  | 0.20±0.02 |  | 0.20±0.01 |  | 0.21±0.02 |  |
|  | IC | Control (n=14) | 0.17±0.02 | ns | 0.17±0.01 | ns | 0.17±0.01 | ns | 0.19±0.01 | ns |
|  |  | Stress (n=13) | 0.17±0.02 |  | 0.18±0.02 |  | 0.17±0.02 |  | 0.18±0.02 |  |
|  | Tha | Control (n=14) | 0.20±0.02 | ns | 0.22±0.01 | ns | 0.22±0.02 | ns | 0.22±0.02 | ns |
|  |  | Stress (n=13) | 0.20±0.02 |  | 0.21±0.01 |  | 0.22±0.02 |  | 0.22±0.02 |  |
|  | BG | Control (n=14) | 0.20±0.02 | ns | 0.21±0.02 | ns | 0.23±0.02 | ns | 0.22±0.02 | ns |
|  |  | Stress (n=13) | 0.20±0.02 |  | 0.21±0.02 |  | 0.22±0.02 |  | 0.22±0.02 |  |
| MD† | Amy | Control (n=14) | 7.77±0.46 | ns | 7.64±0.46 | ns | 7.54±0.39 | ns | 7.59±0.39 | ns |
|  |  | Stress (n=11) | 7.80±0.37 |  | 7.75±0.45 |  | 7.39±0.27 |  | 7.44±0.29 |  |
|  | IC | Control (n=14) | 7.61±0.29 | ns | 7.46±0.19 | ns | 7.40±0.35 | ns | 7.45±0.36 | ns |
|  |  | Stress (n=13) | 7.61±0.19 |  | 7.38±0.21 |  | 7.31±0.21 |  | 7.33±0.38 |  |
|  | Tha | Control (n=14) | 7.08±0.15 | ns | 7.01±0.19 | ns | 6.84±0.21 | ns | 6.93±0.14 | ns* |
|  |  | Stress (n=13) | 7.13±0.17 |  | 7.05±0.15 |  | 6.92±0.18 |  | 7.05±0.27 |  |
|  | BG | Control (n=14) | 6.66±0.16 | ns | 6.70±0.13 | ns | 6.61±0.19 | ns | 6.63±0.15 | ns |
|  |  | Stress (n=13) | 6.66±0.15 |  | 6.71±0.16 |  | 6.70±0.14 |  | 6.77±0.18 |  |
| AD† | Amy | Control (n=14) | 9.32±0.52 | ns | 9.28±0.57 | ns | 9.13±0.48 | ns | 9.28±0.53 | ns |
|  |  | Stress (n=11) | 9.36±0.47 |  | 9.39±0.53 |  | 8.98±0.31 |  | 9.06±0.39 |  |
|  | IC | Control (n=14) | 8.87±0.38 | ns | 8.73±0.21 | ns | 8.66±0.40 | ns | 8.86±0.43 | ns |
|  |  | Stress (n=13) | 8.88±0.30 |  | 8.66±0.30 |  | 8.57±0.35 |  | 8.66±0.50 |  |
|  | Tha | Control (n=14) | 8.51±0.24 | ns | 8.57±0.23 | ns | 8.43±0.28 | ns | 8.51±0.25 | ns* |
|  |  | Stress (n=13) | 8.61±0.24 |  | 8.60±0.23 |  | 8.49±0.21 |  | 8.67±0.40 |  |
|  | BG | Control (n=14) | 8.08±0.31 | ns | 8.24±0.17 | ns | 8.27±0.31 | ns | 8.26±0.28 | ns |
|  |  | Stress (n=13) | 8.05±0.26 |  | 8.21±0.20 |  | 8.30±0.24 |  | 8.42±0.32 |  |
| RD† | Amy | Control (n=14) | 7.00±0.46 | ns | 6.81±0.42 | ns | 6.74±0.37 | ns | 6.74±0.35 | ns |
|  |  | Stress (n=11) | 7.03±0.34 |  | 6.93±0.44 |  | 6.59±0.27 |  | 6.63±0.27 |  |
|  | IC | Control (n=14) | 6.98±0.26 | ns | 6.82±0.19 | ns | 6.76±0.33 | ns | 6.75±0.32 | ns |
|  |  | Stress (n=13) | 6.98±0.19 |  | 6.73±0.20 |  | 6.68±0.17 |  | 6.66±0.33 |  |
|  | Tha | Control (n=14) | 6.36±0.15 | ns | 6.23±0.19 | ns | 6.05±0.22 | ns | 6.14±0.14 | ns* |
|  |  | Stress (n=13) | 6.38±0.16 |  | 6.27±0.14 |  | 6.13±0.20 |  | 6.24±0.23 |  |
|  | BG | Control (n=14) | 5.95±0.12 | ns | 5.93±0.15 | ns | 5.78±0.17 | ns | 5.81±0.14 | 0.042^⁋^ |
|  |  | Stress (n=13) | 5.97±0.13 |  | 5.96±0.18 |  | 5.91±0.15 |  | 5.94±0.17 |  |
| Volume^§^ | Amy | Control (n=14) | 10.93±1.17 | ns | 11.27±1.06 | ns | 11.64±0.91 | ns | 11.28±0.88 | ns |
|  |  | Stress (n=12) | 11.63±1.24 |  | 11.71±1.00 |  | 11.91±1.02 |  | 11.86±1.02 |  |
|  | IC | Control (n=14) | 25.34±3.05 | 0.003 | 25.11±2.49 | ns* | 26.38±4.04 | ns | 27.15±3.33 | ns |
|  |  | Stress (n=12) | 28.83±2.07 |  | 27.09±3.47 |  | 29.18±3.07 |  | 28.29±2.54 |  |
|  | Tha | Control (n=14) | 56.48±6.31 | ns | 57.43±7.19 | ns* | 58.50±6.47 | ns | 59.32±7.55 | ns |
|  |  | Stress (n=12) | 58.75±4.97 |  | 59.22±3.72 |  | 59.84±4.56 |  | 59.87±5.09 |  |
|  | BG | Control (n=14) | 68.45±5.72 | ns | 68.39±5.24 | ns | 71.82±7.92 | ns* | 72.38±6.56 | ns |
|  |  | Stress (n=11) | 67.40±5.12 |  | 70.44±3.72 |  | 71.10±2.86 |  | 72.80±5.67 |  |
| Note: A few subjects were excluded from the analysis when assumptions like no significant outliers or normality were not met.  Amy: amygdala; IC: inferior colliculus; Tha: thalamus; BG: basal ganglia  †Values are expressed in units of ×10^-4^ mm^2^/s.  ^§^Values are expressed in units of mm^3^ without scaling.  *Welch corrected p-values.  ⁋Without or with marginally significant between group difference assessed by mixed design ANOVA. | | | | | | | | | | |
